# Voxel-based structural magnetic resonance imaging (MRI) study of patients with early onset schizophrenia

**DOI:** 10.1186/1744-859X-7-25

**Published:** 2008-12-22

**Authors:** Yujiro Yoshihara, Genichi Sugihara, Hideo Matsumoto, John Suckling, Katsuhiko Nishimura, Takao Toyoda, Haruo Isoda, Kenji J Tsuchiya, Kiyokazu Takebayashi, Katsuaki Suzuki, Harumi Sakahara, Kazuhiko Nakamura, Norio Mori, Nori Takei

**Affiliations:** 1 Department of Psychiatry and Neurology, Hamamatsu University School of Medicine, Hamamatsu, Japan; 2 Osaka-Hamamatsu Joint Research Center for Child Mental Development, Hamamatsu, Japan; 3 Department of Psychiatry and Behavioral Sciences, Tokai University School of Medicine, Isehara, Japan; 4 Brain Mapping Unit, Department of Psychiatry, University of Cambridge, Addenbrooke's Hospital, Cambridge, UK; 5 Department of Radiology, Hamamatsu University School of Medicine, Hamamatsu, Japan; 6 Division of Psychological Medicine, Institute of Psychiatry, King's College, University of London, London, UK

## Abstract

**Background:**

Investigation into the whole brain morphology of early onset schizophrenia (EOS) to date has been sparse. We studied the regional brain volumes in EOS patients, and the correlations between regional volume measures and symptom severity.

**Methods:**

A total of 18 EOS patients (onset under 16 years) and 18 controls matched for age, gender, parental socioeconomic status, and height were examined. Voxel-based morphometric analysis using the Brain Analysis Morphological Mapping (BAMM) software package was employed to explore alterations of the regional grey (GM) and white matter (WM) volumes in EOS patients. Symptoms were assessed using the Positive and Negative Syndrome Scale (PANSS).

**Results:**

EOS patients had significantly reduced GM volume in the left parahippocampal, inferior frontal, and superior temporal gyri, compared with the controls. They also had less WM volume in the left posterior limb of the internal capsule and the left inferior longitudinal fasciculus. The positive symptom score of PANSS (higher values corresponding to more severe symptoms) was negatively related to GM volume in the bilateral posterior cingulate gyrus. The negative symptom score was positively correlated with GM volume in the right thalamus. As for the association with WM volume, the positive symptom score of PANSS was positively related to cerebellar WM (vermis region), and negatively correlated with WM in the brain stem (pons) and in the bilateral cerebellum (hemisphere region).

**Conclusion:**

Our findings of regional volume alterations of GM and WM in EOS patients coincide with those of previous studies of adult onset schizophrenia patients. However, in brain regions that had no overall structural differences between EOS patients and controls (that is, the bilateral posterior cingulate gyrus, the right thalamus, the cerebellum, and the pons), within-subject analysis of EOS patients alone revealed that there were significant associations of the volume in these areas and the symptom severity. These findings suggest that at an early stage of the illness, especially for those with onset before brain maturation, a wide range of disturbed neural circuits, including these brain regions that show no apparent morphological changes, may contribute to the formation of the symptomatology.

## Background

Schizophrenia is a mental disorder with typical onset in early adulthood [1]. Although the disorder has also been identified in children and early adolescents, such occurrences are rare [2,3]. Several studies have gathered and scrutinised brain samples from early onset schizophrenia (EOS) patients in various domains. In particular, since brain morphological abnormalities are consistently found in the general samples of patients with schizophrenia (that is, adult onset populations), researchers have focused on the exploration of brain morphology in patients with EOS, defined herein as schizophrenia with onset under age 18 [4-6]. Using magnetic resonance imaging (MRI), some research groups have reported enlargement of the lateral ventricles [7,8], and regional volume reduction in the superior temporal gyrus [9,10], thalamus [8,11,12], and frontal lobe [12-14] in EOS patients, mirroring findings reported in individuals with adult onset schizophrenia (AOS) [15-17]. However, compared with AOS studies, the number of EOS studies, especially by separate research groups, is still small, and thus studies using an independent sample of EOS patients are in demand.

Previous studies of AOS patients have reported the relationships between structural brain volume alterations, and positive and negative symptoms in various brain regions, such as the superior temporal gyrus [18-20], insula [21], fusiform gyrus [20], parahippocampal gyrus [22], basal ganglia [23], and prefrontal gyri [24]. As for EOS patients, a few studies have investigated the relationships between regional brain changes and the symptom severity, and the regions found to be related to the symptoms in EOS patients, such as the hippocampal [5], occipital, and parietal cortices [25], are not entirely consistent with the reports on AOS patients.

Brain development in early life is thought to be dynamic, with the patterns of growth being diverse across different brain regions [26]. In particular, brain regions that are inherently linked with the pathology of schizophrenia may undergo disproportional changes during the vulnerable period of brain development (that is, the period before adolescence) [27,28]. In the present study, we conducted voxel-based structural MRI analyses to explore any pattern of regional brain tissue volume abnormalities, and to elucidate the relationships between the regional brain volume and the severity of clinical symptoms in EOS patients.

## Methods

### Recruitment of participants

Patients who fulfilled Diagnostic and Statistical Manual of Mental Disorders, 4th edition (DSM-IV) [29] criteria for schizophrenia and who had been under the age of 18 years at onset [4-6] were recruited from among inpatient and outpatient facilities at the Hamamatsu University Hospital and associated hospitals in the city of Hamamatsu, Japan. In all, 18 patients with onset equal to or below 16 years participated in the study. A total of 18 healthy control participants were recruited from the community in Hamamatsu by way of posted advertisement and word of mouth. They were matched to the patients for age, gender, parental socioeconomic status, and height. Participants in both the EOS and control groups were excluded if they had: (1) any current neurological disorders or family history of hereditary neurological disorders, (2) a history of head injury resulting in loss of consciousness, (3) alcohol or substance abuse, or (4) metallic objects in their body (exclusion criterion for MRI). Patients were also excluded if they had any comorbid DSM-IV axis I disorder. The study was approved by the Ethics Committee of the Hamamatsu University School of Medicine. After a complete description of the study to each participant and his or her parents, written informed consent was obtained.

### Clinical assessments

Diagnosis was made on the basis of interviews by two trained psychiatrists (one of whom was a child psychiatrist), along with reference to medical records and information from family members and attending physicians. The dose and duration of medication were also recorded. Age at onset of schizophrenia was defined as the age when patients first clearly manifested either delusions, hallucinations, or thought disorders. Psychopathology was assessed with the Positive and Negative Syndrome Scale (PANSS) [30]. Diagnosis was established with the Structured Clinical Interview for DSM-IV (SCID) [31]. For those participants under the age of 16, the interview was supplemented by the KID SCID [32]. Parental socioeconomic status was determined according to the Standard Occupational Classification [33], and handedness was determined by self-report. Statistical significance was set at p < 0.05 for clinical comparisons, and for the volume measures (two-tailed).

### MRI acquisition

All participants were scanned with a GE Signa 1.5-T system (GE Medical Systems, Milwaukee, WI, USA) at the Hamamatsu University School of Medicine. A preliminary localising scan in the coronal plane was used to identify anterior and posterior commissures, and to prescribe acquisition of a dual echo fast spin echo dataset in a plane parallel to the intercommissural line. Contiguous, interleaved proton density- and T2-weighted images, each 3-mm thick, were obtained to provide whole brain coverage. The repetition time (TR) was 4000 ms, and echo times (TE) were 14 and 84 ms with an 8-echo train length. The matrix size was 256 × 192 collected from a rectangular field of view of 24 cm × 18 cm, giving an in-plane resolution of 0.859 mm. The total acquisition time was 9 min 36 s.

### Data analysis

Group differences in grey and white matter were assessed using the Brain Analysis Morphological Mapping (BAMM) software package . This process has previously been described in detail for both adult and child populations [23,34,35]. Briefly, images were processed to remove extracerebral tissues [36], and then segmented into grey and white matter, cerebrospinal fluid (CSF) and a fourth class including dura, vessels, and other extraneous tissues which were subsequently ignored [37]. Global volumes of grey and white matter, CSF, and whole brain (grey and white matter plus CSF) were calculated and compared across groups using independent t tests with the level of significance set at p < 0.05. The segmented images were mapped into the standard space of Talairach and Tournoux [38] by minimising the sum of the square intensity differences between each proton density image and a template and applying the derived mappings to the segmented tissue maps. All maps were smoothed with a Gaussian kernel of 2 mm standard deviation [37]. Between-group differences in grey matter volume and white matter volume were estimated by fitting an analysis of covariance (ANCOVA) model at each intracerebral voxel in standard space, which included the age at scan, sex, and whole brain volume as covariates. Maps of the appropriate normalised coefficient were subject to an inference procedure in which the significance of a three-dimensional cluster statistic was assessed using non-parametric methods [39]. The statistical thresholds were corrected for multiple comparisons by controlling the 'family-wise error rate', in this case by setting the p value used such that < 1 false-positive cluster was expected under the null hypothesis [39]. A cluster of grey or white matter abnormality was defined as a deficit or an excess depending on whether the volume was reduced or increased in the EOS group relative to the control group. Within the patient group, the relationships between grey and white matter volume and positive, negative and global score (PANSS) were estimated by fitting a regression model at each intracerebral voxel in standard space for each tissue class separately.

When the assumptions of the parametric methods are not guaranteed, the non-parametric methods provide the only analysis that can be considered valid and exact. As the distribution of the structural data derived from MRI scans may violate the assumptions, such as normal distribution, we thus employed non-parametric methods in this study.

## Results

### Demographic and clinical characteristics

The EOS and healthy control groups were similar as regards the distribution of age, sex, ethnicity, social class, and height (Table 1). All participants were right-handed. Healthy controls had a significantly (p < 0.001) higher mean IQ than the patients. In all, 17 EOS patients had received antipsychotic medication, and 1 EOS patient had never received antipsychotic medication.

**Table 1:** Subject characteristics

**Variable**	**Healthy controls** **(n = 18)**	**EOS patients** **(n = 18)**	**p Value**
Age (years)^a^	15.8 (1.3)	15.8 (1.8)	0.693
Sex (male/female)^b^	9/9	9/9	1.00
Social class 1–3^b^	16 (89%)	14 (78%)	0.371
Height (cm)^a^	163.6 (7.1)	158.8 (8.6)	0.085
IQ^a^	97.3 (11.8)	72.8 (15.3)	0.001
Duration of illness (years)	-	1.2 (0.9)	-
Positive and Negative Syndrome Scale:			
Positive score	-	13.8 (4.4)	-
Negative score	-	19.1 (10.0)	-
Global score	-	31.1 (10.7)	-

Values are given as the mean (SD), except for sex and social class.

^a^Two-tailed t test; ^b^χ^2 ^test.

EOS, early onset schizophrenia; SD, standard deviation.

### Brain and CSF volumetric measures

Global volumes for the whole brain and each of the three main tissue classes (grey matter, white matter, and CSF) are shown in Table 2. In the EOS group, grey matter (GM) volume was 5.5% smaller and white matter (WM) volume was 3.9% smaller than in the control group, whereas CSF volume in the EOS group was 11.5% larger than in the control group. The differences in GM and CSF between the two groups were significant (p < 0.032 and 0.008, respectively), but the difference in WM was not (p < 0.12). In addition, EOS patients had a significantly (4.0%; p < 0.009) smaller GM to whole brain ratio and significantly (15.4%; p < 0.001) larger CSF to whole brain ratio compared with the controls.

**Table 2:** Global brain volumes

**Global brain volumes**	**Control group**^**a**^ **(n = 18)**	**EOS group**^**a**^ **(n = 18)**	**Group difference** **(%)**	**t**	**p Value**
Whole brain (ml)	1,325.7 (106.4)	1,290.0 (47.4)	2.7 (Control > EOS)	1.302	0.205
Grey matter (ml)	657.2 (60.4)	621.3 (29.5)	5.5 (Control > EOS)	2.271	0.032
White matter (ml)	496.5 (46.9)	476.9 (19.7)	3.9 (Control > EOS)	1.638	0.115
CSF (ml)	172.0 (20.3)	191.8 (21.9)	11.5 (EOS > Control)	-2.813	0.008
Grey matter/whole brain ratio	0.50 (0.01)	0.48 (0.02)	4.0 (Control > EOS)	2.814	0.009
White matter/whole brain ratio	0.37 (0.01)	0.37 (0.01)	0	1.314	0.199
CSF/whole brain ratio	0.13 (0.02)	0.15 (0.01)	15.4 (EOS > Control)	-3.507	0.001

^a^Values are given as the mean (SD).

CSF, cerebrospinal fluid; EOS, early onset schizophrenia; SD, standard deviation.

### Regional grey matter (GM) and white matter (WM) alterations in EOS patients

**Figure 1:**
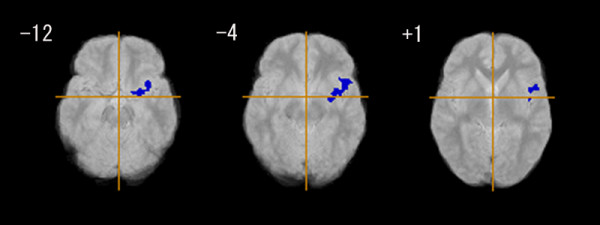
**Grey matter regional differences between early onset schizophrenia group (n = 18) and control group (n = 18)**. Blue regions denote areas of grey matter deficits in the early onset schizophrenia group relative to the control group. The left side of the figure represents the right side of the brain; the z coordinate for each axial slice in the standard space of Talairach and Tournoux [38] is given in mm. Cluster-wise probability of type I error: p = 0.001, with less than one false-positive cluster expected over the whole map.

**Figure 2:**
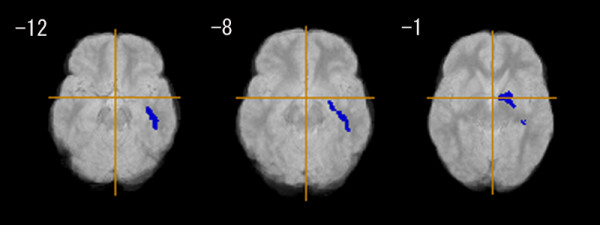
**White matter regional differences between early onset schizophrenia group (n = 18) and control group (n = 18)**. Blue regions denote areas of white matter deficits in the early onset schizophrenia group relative to the control group. The left side of the figure represents the right side of the brain; the z coordinate for each axial slice in the standard space of Talairach and Tournoux [38] is given in mm. Cluster-wise probability of type I error: p = 0.001, with less than one false-positive test expected over the whole map.

The EOS group had a significantly smaller GM in the left parahippocampal gyrus (Brodmann's area: 34), the left inferior frontal gyrus (Brodmann's area: 47), and the left superior temporal gyrus (Brodmann's area: 22), compared with the control group (Figure 1, Table 3). The EOS group also had significantly less WM in the left posterior limb of the internal capsule, and the left inferior longitudinal fasciculus (Figure 2, Table 3).

**Table 3:** Grey and white matter regional differences between early onset schizophrenia (EOS) group and control group

**Area**	**Brodmann area**	**Talairach coordinate of centroid voxel (mm)**			**Number of voxels in cluster**
			
		**x**	**y**	**z**	
Control > EOS in grey matter:					
Left parahippocampal gyrus,	34/47/22	-57	2	-4	423
Left inferior frontal gyrus,					
Left superior temporal gyrus					
Control > EOS in white matter:					
Left posterior limb of internal capsule		-20	-8	-8	418
Left inferior longitudinal fasciculus					

### Association between symptoms and volumetric measures (GM and WM) in EOS patients

**Figure 3:**
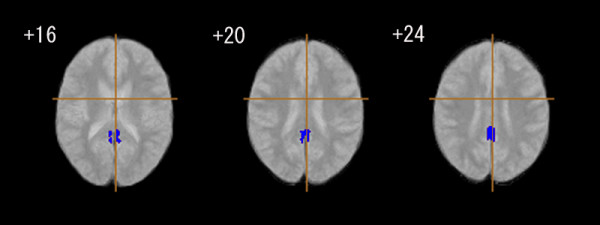
**Association between positive syndrome scores and grey matter tissue class volume in patients with early onset schizophrenia (n = 18)**. Blue regions denote areas in which lower grey matter volume is predicted by a higher score on a positive symptom rating scale in the early onset schizophrenia group. The left side of the figure represents the right side of the brain; the z coordinate for each axial slice in the standard space of Talairach and Tournoux [38] is given in mm. Cluster-wise probability of type I error: p = 0.001, with less than one false-positive test expected over the whole map.

**Figure 4:**
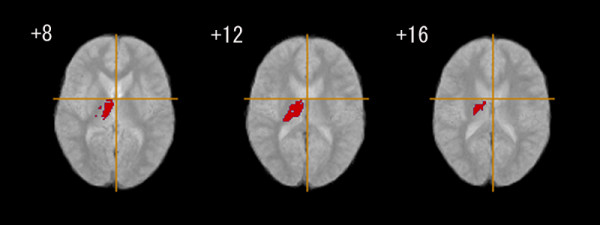
**Association between negative syndrome scores and grey matter tissue class volume in patients with early onset schizophrenia (n = 18)**. Red regions denote areas in which larger grey matter volume is predicted by a high score on a negative symptom rating scale in the early onset schizophrenia group. The left side of the figure represents the right side of the brain; the z coordinate for each axial slice in the standard space of Talairach and Tournoux [38] is given in mm. Cluster-wise probability of type I error: p = 0.001, with less than one false-positive test expected over the whole map.

**Figure 5:**
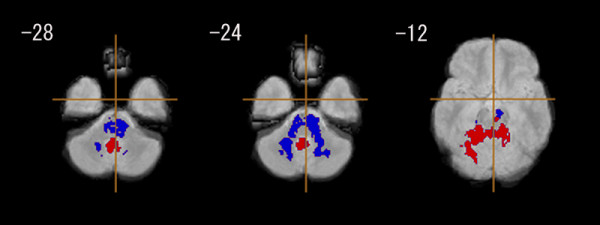
**Association between positive/negative syndrome scores and white matter tissue class volume in patients with early onset schizophrenia (n = 18)**. Red regions denote areas in which larger white matter volume is predicted by a higher score on a positive symptom rating scale in the early onset schizophrenia group. Blue regions denote areas in which lower white matter volume is predicted by a higher score on a positive symptom rating scale in the early onset schizophrenia group. The left side of the figure represents the right side of the brain; the z coordinate for each axial slice in the standard space of Talairach and Tournoux [38] is given in mm. Cluster-wise probability of type I error: p = 0.001, with less than one false-positive test expected over the whole map.

The positive symptom score of PANSS (with higher values in the PANSS corresponding to more severe symptoms) was negatively related to GM volume in the bilateral posterior cingulate gyrus (Figure 3, Table 4). The negative symptom score was positively correlated with GM volume in the right thalamus (Figure 4, Table 4). As for the association with WM volume, the positive symptom score of PANSS was positively related to cerebellar WM (vermis region), and negatively correlated with WM in the brain stem (pons) and in the bilateral cerebellum (hemisphere region) (Figure 5, Table 4). We found no significant association between regional WM volume change and the negative symptom score of PANSS.

**Table 4:** Association between positive/negative syndrome scores and grey and white matter tissue class volume in patients with early onset schizophrenia (n = 18)

**Tissue class and relative difference region**	**Talairach coordinate of centroid voxel (mm)**			**Number of voxels in cluster**
		
	**x**	**y**	**z**	
Grey matter:				
Positive symptom				
Negative correlation				
Bilateral posterior cingulate gyrus	1	-55	20	256
Negative symptom				
Positive correlation				
Right thalamus	9	-14	12	224
				
White matter:				
Positive symptom				
Positive correlation				
Cerebellum (vermis)	8	-48	-12	1,370
Negative correlation				
Brain stem (pons)	-3	-21	-24	921
Cerebellum (hemisphere)				

## Discussion

To our knowledge, this is the first voxel-based morphometry study indicating significant relationships between regional brain volume alterations and clinical symptoms in EOS patients (that is, with onset under 16 years). We found a significant GM volume reduction and a significant increase of CSF in EOS patients. The EOS patients had a significant reduction of regional GM in the left parahippocampal gyrus, the left inferior frontal gyrus, and the left superior temporal gyrus. In addition, they had reduced regional WM in the left posterior limb of the internal capsule, and in the left inferior longitudinal fasciculus. When the correlations between regional GM volume and positive symptoms were examined within the EOS patient group, positive symptoms were found to be significantly correlated with the reduced GM volume in the bilateral posterior cingulate gyrus, with the increased WM volume in the cerebellum (vermis region), and with the reduced WM volume in the brain stem and the bilateral cerebellum (hemisphere region). We also found a relationship between the severity of negative symptoms and the increased GM volume in the right thalamus.

The findings of a GM volume reduction and CSF increase in individuals with EOS in this study are compatible with previous volumetric studies of both EOS and adult onset schizophrenia (AOS) patients [7,13,28,40-42], suggesting that the pattern of volumetric alterations (that is, GM volume reduction and CSF increase) may be an inherent feature of schizophrenia irrespective of age at onset. The GM volume reduction in schizophrenia as a whole could reflect an exaggeration of a normal maturational process of synaptic/dendritic pruning during adolescence [28].

The voxel-based analysis revealed that in the EOS group, the regional volume reduction of GM was evident in the left parahippocampal gyrus, the left inferior frontal gyrus, and the left superior temporal gyrus. These findings were consistent with those of the previous studies in patients with AOS [15,43-45]. However, because regional grey matter alterations have frequently been observed in various brain regions of AOS patients, rather than being limited to these three brain regions alone [15], and because grey matter abnormalities are deemed to be a fundamental part of the pathophysiology of schizophrenia, the observation of grey matter changes in these three regions (that is, the parahippocampal, inferior frontal, and superior temporal gyrus) in individuals with EOS in the present study may indicate that the disease originates in these areas. Another possible explanation is that these three regions may be more subject to brain insults that are related to the predisposition to schizophrenia.

We also found white matter morphological changes in EOS patients. The reduction of WM volume was located in the left posterior limb of the internal capsule, and the left inferior longitudinal fasciculus. Although no prior study has addressed the regional WM volume alterations in EOS, the current findings are similar to those reported by Sigmundsson *et al*. [23], who investigated WM volume changes in AOS patients. In addition, diffusion tensor imaging (DTI) studies of AOS [46] and EOS [47] patients have also shown WM abnormalities in similar regions. Therefore, there is evidence emerging from a variety of sources to suggest that WM abnormalities are part of the patterns of brain parenchymal aberration associated with schizophrenia, although they may be a secondary process. In addition, we found a relationship between the brain stem (pons) volume reduction and positive symptoms. This may be intriguing in view of the evidence, from a positron emission tomography (PET) study, showing dysregulation of dopaminergic transmission in the midbrain in schizophrenia [48].

In our sample of EOS patients, the brain region that was found to be associated with the severity of the positive symptoms was the posterior cingulate gyrus. Although similar findings have been reported in AOS patients [49], most AOS studies have shown an association with other brain regions, such as the superior temporal gyrus [18-20], insula [21], fusiform gyrus [20], and parahippocampal gyrus [22]. It is of note, however, that the region of the posterior cingulate gyrus has also been reported to be associated with the positive symptoms in other neuroimaging studies of patients with AOS, such as pharmacological functional MRI [50] and positron emission tomography (PET) studies [51]. Furthermore, additional evidence from some AOS studies that the posterior cingulate gyrus volume reduction is associated with other clinical features, such as poor clinical outcome [52], and the lack of insight and judgment [53], suggests that neural circuits involving the posterior cingulate gyrus may have an important role in the pathophysiology of schizophrenia. Given that the posterior cingulate is related to processing of emotionally salient stimuli and spatial attention [54], our finding that the posterior cingulate gyrus became prominent in relation to positive symptoms suggests that this region may be involved in the formation of delusions in individuals with a very early age of onset and at an early stage of the illness.

In the present study, the severity of the positive symptoms of schizophrenia was found to be associated with increased WM volume in the cerebellum (vermis region) and with decreased WM volume in the cerebellum (hemisphere region). The finding in the cerebellar vermis region is consistent with that of a previous AOS study [55] showing a positive correlation between vermis WM volume and the severity of positive symptoms. As regards the cerebellar hemisphere region, neither previous studies employing EOS patients nor those with AOS patients have reported significant correlations with positive symptoms. This is the first study, to our knowledge, to demonstrate a relationship between positive symptoms and a decreased WM volume of the cerebellar hemisphere region.

The severity of the negative symptoms was found to be associated with the increased GM volume of the right thalamus. This finding is similar to the results of prior AOS studies showing a positive correlation between thalamic volume and the severity of negative symptoms [56-58]. The thalamus is thought to play a role in sensory gating, a disruption of which has been reported to be involved in schizophrenia [59,60]. Previous AOS studies have also reported correlations between the negative symptoms and structural alterations in other brain regions: namely, reduced volume in the fusiform gyrus [19], frontal lobe WM [24,61,62], prefrontal GM [63], cingulate WM and internal capsule [64], and increased volume in the posterior superior temporal gyrus [65]. However, in our sample of EOS patients, there were no associations between morphological measures in any of these regions and the severity score of negative symptoms. It could be that at an early stage of brain development (that is, in adolescence), the thalamus, which has reciprocal connectivity with the frontal regions, may be predominantly involved in generating negative symptoms (that is, cognitive deficits) via disturbed connectivity.

A question may arise as to why no relationships were evident between the clinical symptoms and the morphological measures in the three main brain regions (the frontal, temporal, and parahippocampal gyri) showing significant volume reductions in our EOS patients. What is puzzling is that other regions (that is, the posterior cingulate gyrus and the thalamus) were found to be associated with the symptoms in this study. One possible interpretation is that disturbed neural circuits rather than structural alterations *per se *may play a role in the formation of symptoms in early onset schizophrenia patients (that is, patients with onset prior to brain maturation). Impaired circuits may involve interconnections between the posterior cingulate gyrus and the temporal lobe [66] and between the thalamus and frontal lobe [67,68].

The results of this study should be interpreted in the context of the following limitations. First, the number of subjects was relatively small. In spite of this, the fact that we were able to detect the regional brain volume alterations in a unique sample of patients with EOS and the finding that most of the abnormalities found were identical to those for the general population of adult onset schizophrenia patients, may support the robustness of the current findings. Second, IQ score was not matched between the case and control groups; that is, the mean IQ was significantly lower in the patients than in the controls. Thus, we conducted an additional analysis in which IQ was adjusted for as a covariate, and found that the regional brain volume differences (for example, the three main GM regions) between the groups remained significant. As a result, the effects of IQ on the findings can be taken as minimal, especially with respect to the regional brain changes. Third, the effect of medication was not considered when regional brain changes were compared between the case and control groups. Antipsychotic medication can affect regional brain morphology in schizophrenia, particularly in the thalamus [69] and basal ganglia [70], resulting in increased volumes in these regions. If physicians tended to administer greater dosages of antipsychotic medication to combat the negative symptoms, then the relationship between increased volume in the thalamus and the negative symptoms found in this study would be accounted for by the medication effect. We performed an analysis in which medication dose was entered as a covariate, and found that the correlation between the severity of negative symptoms and the increase of volume in the thalamus remained significant.

Pathophysiological changes in schizophrenia – including brain morphological changes – may be drastic, especially in schizophrenic patients with onset before brain maturation. Therefore, studying schizophrenia patients whose age of onset is as early as childhood is valuable in clarifying the pathophysiological dynamics of the disorder. Large-scale longitudinal studies are also needed to elucidate brain morphological changes in young populations with early onset schizophrenia.

## Competing interests

The authors declare that they have no competing interests.

## Authors' contributions

YY, GS, and TT designed the study. YY and TT contributed to recruitment of study subjects and MRI data collection. HM and TT were involved in clinical evaluation of the participants and procedures of the MRI data acquisition. JS provided assistance of the MRI data collection and performed data analyses. KNi, HI, KJT, KT, KS, HS, KNa and NM participated in the stage of designing the study and recruitment of the study subjects. NT supervised the study and refined the analyses. YY, GS, and NT wrote the manuscript. All the authors read the paper and approved the final form of the manuscript.
